# Hydrogenotrophic Microbial Reduction of Oxyanions With the Membrane Biofilm Reactor

**DOI:** 10.3389/fmicb.2018.03268

**Published:** 2019-01-10

**Authors:** Chen Zhou, Aura Ontiveros-Valencia, Robert Nerenberg, Youneng Tang, David Friese, Rosa Krajmalnik-Brown, Bruce E. Rittmann

**Affiliations:** ^1^Biodesign Swette Center for Environmental Biotechnology, Arizona State University, Tempe, AZ, United States; ^2^Tecnologico de Monterrey, Escuela de Ingenieria y Ciencias, Campus Puebla, Puebla, Mexico; ^3^Department of Civil & Environmental Engineering & Earth Sciences, University of Notre Dame, Notre Dame, IN, United States; ^4^Department of Civil and Environmental Engineering, FAMU-FSU College of Engineering, Florida State University, Tallahassee, FL, United States; ^5^APTwater LLC, Pittsburg, CA, United States

**Keywords:** oxyanions, hydrogen, membrane biofilm reactor (MBfR), surface loading, microbial ecology

## Abstract

Oxyanions, such as nitrate, perchlorate, selenate, and chromate are commonly occurring contaminants in groundwater, as well as municipal, industrial, and mining wastewaters. Microorganism-mediated reduction is an effective means to remove oxyanions from water by transforming oxyanions into harmless and/or immobilized forms. To carry out microbial reduction, bacteria require a source of electrons, called the electron-donor substrate. Compared to organic electron donors, H_2_ is not toxic, generates minimal secondary contamination, and can be readily obtained in a variety of ways at reasonable cost. However, the application of H_2_ through conventional delivery methods, such as bubbling, is untenable due to H_2_'s low water solubility and combustibility. In this review, we describe the membrane biofilm reactor (MBfR), which is a technological breakthrough that makes H_2_ delivery to microorganisms efficient, reliable, and safe. The MBfR features non-porous gas-transfer membranes through which bubbleless H_2_ is delivered on-demand to a microbial biofilm that develops naturally on the outer surface of the membranes. The membranes serve as an active substratum for a microbial biofilm able to biologically reduce oxyanions in the water. We review the development of the MBfR technology from bench, to pilot, and to commercial scales, and we elucidate the mechanisms that control MBfR performance, particularly including methods for managing the biofilm's structure and function. We also give examples of MBfR performance for cases of treating single and co-occurring oxyanions in different types of contaminated water. In summary, the MBfR is an effective and reliable technology for removing oxyanion contaminants by accurately providing a biofilm with bubbleless H_2_ on demand. Controlling the H_2_ supply in accordance to oxyanion surface loading and managing the accumulation and activity of biofilm are the keys for process success.

## Introduction

As documented in other articles in this issue, many water contaminants are oxyanions (Kumar et al., 2014; Yin et al., 2018). Sources include agriculture, mining, metal-working and aerospace industries, municipal wastewater, and natural minerals. The impacts to human and ecosystem health are wide-ranging and can be severe. The risks of some oxyanions have been well-recognized for years, and governments have established maximum contaminant levels (MCLs), such as 10 mg N/L for nitrate (NO${}_{3}^{-}$), 1 mg N/L for nitrite (NO${}_{2}^{-}$), 0.1 mg Cr/L for chromate (CrO${}_{4}^{2-}$), and 0.05 mg Se/L for selenate (SeO${}_{4}^{2-}$) (USEPA, 2012). Other oxyanions are emerging contaminants and do not yet have MCLs or discharge standards.

An important feature of oxyanion contamination is that the oxyanions often occur in mixtures having wide-ranging concentrations. For example, μg/L levels of perchlorate (ClO${}_{4}^{-}$) often are present alongside mg N/L levels of NO${}_{3}^{-}$ in groundwater (Nerenberg et al., 2002, 2008; Kimbrough and Parekh, 2007). Coal-mining wastewaters contain mg/L concentrations of selenate (SeO${}_{4}^{2-}$) alongside sulfate (SO${}_{4}^{2-}$) at hundreds to thousands mg/L (Twidwell et al., 1999; Kløve, 2001). Flue-gas desulfurization (FGD) wastewater contains SeO${}_{4}^{2-}$ and SO${}_{4}^{2-}$ along with NO${}_{3}^{-}$ (Van Ginkel et al., 2011b).

Compared to physical and chemical approaches, mainly including adsorption (Hiemstra and Van Riemsdijk, 1999; Cumberland and Strouse, 2002; Peak, 2006) and catalytic reduction (Chaplin et al., 2012; Yin et al., 2018), microbiological reduction is an effective, economic, and sustainable means to simultaneously remove oxyanions from water. In microbial reduction, the oxyanion regularly functions as a respiratory electron acceptor for bacteria (Rittmann and McCarty, 2001; Martin and Nerenberg, 2012; Madigan et al., 2014; Rittmann, 2018). In respiration, electrons (*e*^−^) are transferred to the acceptor by their movement along a membrane-bound electron-transport chain that exports protons (H^+^) to the outside of the membrane. This sets up a H^+^ concentration gradient across the membrane, which creates a proton motive force. The transport of H^+^ across the membrane in parallel with the proton motive force leads to the production of ATP, the energy currency that organisms use to drive biomass synthesis and maintenance. Thus, respiratory reduction of oxyanions helps select for bacteria capable of respiring on it.

A major advantage of microbiological reduction is that, in most cases, the reduced products are either harmless or precipitate as solids. Here are a few examples of reduction half reactions:

(1) $${\text{NO}}_{3}^{-}+6{\text{H}}^{+}+{\text{5e}}^{-}\to \text{0}.{\text{5N}}_{\text{2}}+3{\text{H}}_{2}\text{O}$$

(2) $${\text{ClO}}_{4}^{-}+8{\text{H}}^{+}+8{\text{e}}^{-}\to {\text{Cl}}^{-}+4{\text{H}}_{2}\text{O}$$

(3) $${\text{SeO}}_{4}^{2-}+8{\text{H}}^{+}+6{\text{e}}^{-}\to {\text{Se}}^{0}+4{\text{H}}_{2}\text{O}$$

(4) $${\text{CrO}}_{4}^{2-}+5{\text{H}}^{+}+3{\text{e}}^{-}\to \text{Cr}{\left(\text{OH}\right)}_{3}+{\text{H}}_{2}\text{O}$$

Among the products of these reactions, N_2_ is a harmless gas that evolves from water phases, Cl^−^ is a harmless anion, Se^0^ is insoluble and can be recovered, and Cr(OH)_3_ is a solid formed at neutral to alkaline pH.

A second advantage of microbiological reduction is that it occurs without the need for extreme conditions of temperature or pH. Likewise, no hazardous materials need to be added. Third, the bacteria are natural, self-generating, and adaptive catalysts.

To carry out microbiological reduction of oxyanions, bacteria require an electron-donor substrate, i.e., a source of electrons. An electron-donor substrate possesses two essential characteristics. The first is that it is reduced, which means that it has electron that can be removed, a process called oxidation. The second is that the bacteria have enzymes that allow them to initiate electron removal. Because most waters contaminated by oxyanions lack sufficient electron donor substrates, a donor must be provided externally.

For bacteria, available electron donors fall into two categories: organic and inorganic. An example of an organic donor is acetate (CH_3_COO^−^), which releases 8 e^−^ by this oxidation half reaction: < math>

(5) $${\text{CH}}_{3}{\text{COO}}^{-}+{\text{H}}_{2}\text{O}\to 2{\text{CO}}_{2}+7{\text{H}}^{+}+8e^{-}$$

Bacteria that utilize organic donors like acetate are called heterotrophs, because they also use the organic donor as their carbon source for biomass synthesis. An example of an inorganic electron donor is hydrogen gas (H_2_), and its oxidation half reaction generates 2 electrons:

(6) $${\text{H}}_{2}\to 2{\text{H}}^{+}+2e^{-}$$

Bacteria that utilize H_2_ at the donor usually are mostly autotrophs, which means that they use some of the electrons to reduce CO_2_ to make organic carbon for biomass synthesis. Because full reduction of ClO${}_{4}^{-}$ requires 8 *e*^−^ per mol, we need to deliver at least 1 mol CH_3_COO^−^ or 4 mol H_2_ per mol ClO${}_{4}^{-}$.

What is the best choice for the electron-donor substrate? The decision is based on four criteria: (1) The bacteria that reduce the oxyanion of interest are able to oxidize the donor. (2) The donor is not hazardous. (3) The donor has a reasonable cost. (4) The donor can be obtained and delivered reliably and accurately. The next section details why H_2_ meets these criteria.

## Hydrogen gas (H_2_) as a Universal Electron Donor

Microbial metabolism of H_2_ is dominant in deep soil, aquifers, and sediments (Morita, 1999), and it is associated with hydrogenases, a group of enzymes that usually employ metal co-factors such as iron ([Fe], [FeFe]), nickel ([FeNi]), and/or metalloids ([FeNiSe]). Hydrogenases are able to reversibly catalyze the conversion of H_2_ into two electrons and two protons (Heinekey, 2009):

(7) $${\text{H}}_{2}⇌2{\text{H}}^{+}+2e^{-}$$

The forward reaction provides energy for the organisms by establishing transmembrane proton gradients for ATP production (Vignais et al., 2001), while the backward reaction enables H_2_ evolution or uptake (Lubitz et al., 2014). When mediated by hydrogenases, H_2_ can serve as an electron donor for reducing a wide spectrum of oxidized compounds, including inorganic oxyanions, metals and metalloids, and halogenated organics (Rittmann, 2018). In addition, hydrogenases can function in aerobic and anaerobic conditions (Morita, 1999; Kubas, 2007).

In nature, hydrogenases are ubiquitously present and function in bacteria, archaea, and some eukarya (Lubitz et al., 2014). On the one hand, many heterotrophs contain genes that encode hydrogenases that can be expressed when H_2_ is the sole energy source. On the other hand, some microorganisms capable of H_2_ oxidation also are able to utilize simple organic electron donors and are regarded as facultative chemolithotrophs (Madigan et al., 2012).

When utilizing H_2_ as the sole electron donor, most microorganisms assimilate CO_2_ as their exclusive carbon source, which is known as autotrophic growth. Exceptions to the rule can be found, however. For example, the dechlorinator *Dehalococcoides* (Maymó-Gatell et al., 1997; He et al., 2003) and the sulfate-reducer *Desulfovibrio* (Badziong et al., 1978; Noguera et al., 1998) require carbon derived from acetate:

(8) $$\begin{array}{l} 2{\text{CH}}_{3}{\text{COO}}^{-}+{\text{HCO}}_{3}^{-}+2{\text{H}}_{2}+3{\text{H}}^{+}+{\text{NH}}_{3}\to {\text{C}}_{5}{\text{H}}_{7}{\text{O}}_{2}\text{N}\\ +5{\text{H}}_{2}\text{O} \end{array}$$

Compared to organic and other inorganic electron donors, such as H_2_S and Fe^0^, H_2_ is not toxic, does not generate any secondary contamination, and can be readily obtained in a variety of ways at a reasonable cost (Karanasios et al., 2010). H_2_ gas can be purchased from gas suppliers and stored on site, or it may be generated on site. The most common processes for on-site H_2_ generation are water electrolysis and methane reforming. Electrolysis only requires water and electricity, and a wide range of capacities are commercially available. Methane reforming has been used mainly in larger systems, but smaller systems have become more common in recent years (Schjølberg et al., 2012). An advantage of on-site production is that storage can be minimized to reduce risk.

H_2_ can be supplied to the liquid phase of a bioreactor in a variety of ways (Rezania et al., 2007; Di Capua et al., 2015; Epsztein et al., 2016). For example, H_2_ bubbling (also called sparging) is the simplest method, but its application is limited by the combination of H_2_'s low water solubility and combustibility. With a Henry's constant of 0.00078 mol/kg^*^bar (Sander, 1999), water in equilibrium with 1 atm of H_2_ at 25°C has a concentration of only 1.6 mg/L H_2_. This means that the driving force for H_2_ mass transfer into the water is low unless the partial pressure of H_2_ is far above atmospheric, but a high H_2_ pressure runs the risk of off-gassing, which wastes the H_2_ and could create a combustible atmosphere. Concerning combustibility, the lower and upper limits of combustion are 4 and 75% by volume, respectively (Chatterjee et al., 1999).

Bubble-less delivery of H_2_ can overcome the problems of off-gassing and combustibility. Electrolysis of water, corrosion of iron, and diffusion through non-porous membranes are options for bubble-less delivery. The simplest and most developed approach to bubble-less H_2_ gas transfer is via gas-permeable, non-porous membranes (Martin and Nerenberg, 2012). In this approach, H_2_ is delivered directly to the base of a biofilm, where it can be consumed by the bacteria before reaching the bulk liquid. Key advantages of this approach include achieving close to 100% gas-utilization efficiency, avoiding off-gassing, and a controllable and accurate delivery rate (Tang et al., 2010). The membranes are available in diameters of 50 to 500 μm, allowing high specific surface areas and compact reactors. Unlike with organic donors, overdosing H_2_ is avoided, as its on-demand delivery is based on its uptake in the biofilm (Rittmann, 2018), which transforms low water solubility from a problem to a benefit. Furthermore, the non-porous gas-transfer membranes allow only gases to pass through them; this means that membrane fouling, which is common in water filtration membranes, is not relevant for the MBfR.

No matter the delivery method, care is needed to avoid gas leaks or conditions where a headspace is formed with a mixture of H_2_ and air. Since H_2_ is very light and disperses rapidly and vertically into the atmosphere, appropriate ventilation (either active or passive) and eliminating overhead containment zones are critical, but can be easily designed.

## Focus of the Review

Until recently, H_2_'s low solubility and combustibility thwarted its use as the electron donor for biological reductions in engineered systems. This roadblock has been removed with the development of the Membrane Biofilm Reactor (MBfR), which makes H_2_ delivery to microorganisms efficient, reliable, and safe by delivering H_2_ via bubble-less gas-transfer membranes (Martin and Nerenberg, 2012; Nerenberg, 2016). Bubble-less delivery avoids transfer inefficiency and risk of flammability that are inherent to bubbling the gas into the liquid. Furthermore, the H_2_ is delivered on-demand to a microbial biofilm that develops naturally on the surface of the membranes.

The focus of this review is on oxyanion reduction using bubble-less H_2_ delivery in the MBfR. The review begins by describing the MBfR in terms of its “membrane + biofilm” principles. It explores the unique situation of substrate counter-diffusion in the biofilm, the history of how the MBfR has moved from research to commercialization, and the roles of biofilm management, gas back-diffusion, and the biofilm's microbial ecology. Then, a series of performance case studies describes the ways in which the MBfR can be used to reduce one oxyanion or a mixture of oxyanions.

## The Membrane Biofilm Reactor (MBfR)

### “Membrane + Biofilm” Principles

The first principle of the MBfR is that it delivers H_2_ without forming bubbles. Several types of hollow-fiber membranes can be used in the MBfR; the most common are dense, single-layer polypropylene fibers and composite fibers made of a dense internal layer of polyethylene sandwiched by external layers of macroporous polyurethane. In either case, the key characteristic is that they can be pressurized with H_2_ gas without forming bubbles. This prevents off-gassing and allows H_2_ delivery to be on-demand and 100% efficient. In addition, the use of these dense (non-porous) polymeric gas-permeable membranes in MBfRs provides long-term stable gas transmission without fouling problems.

**Figure 1:**
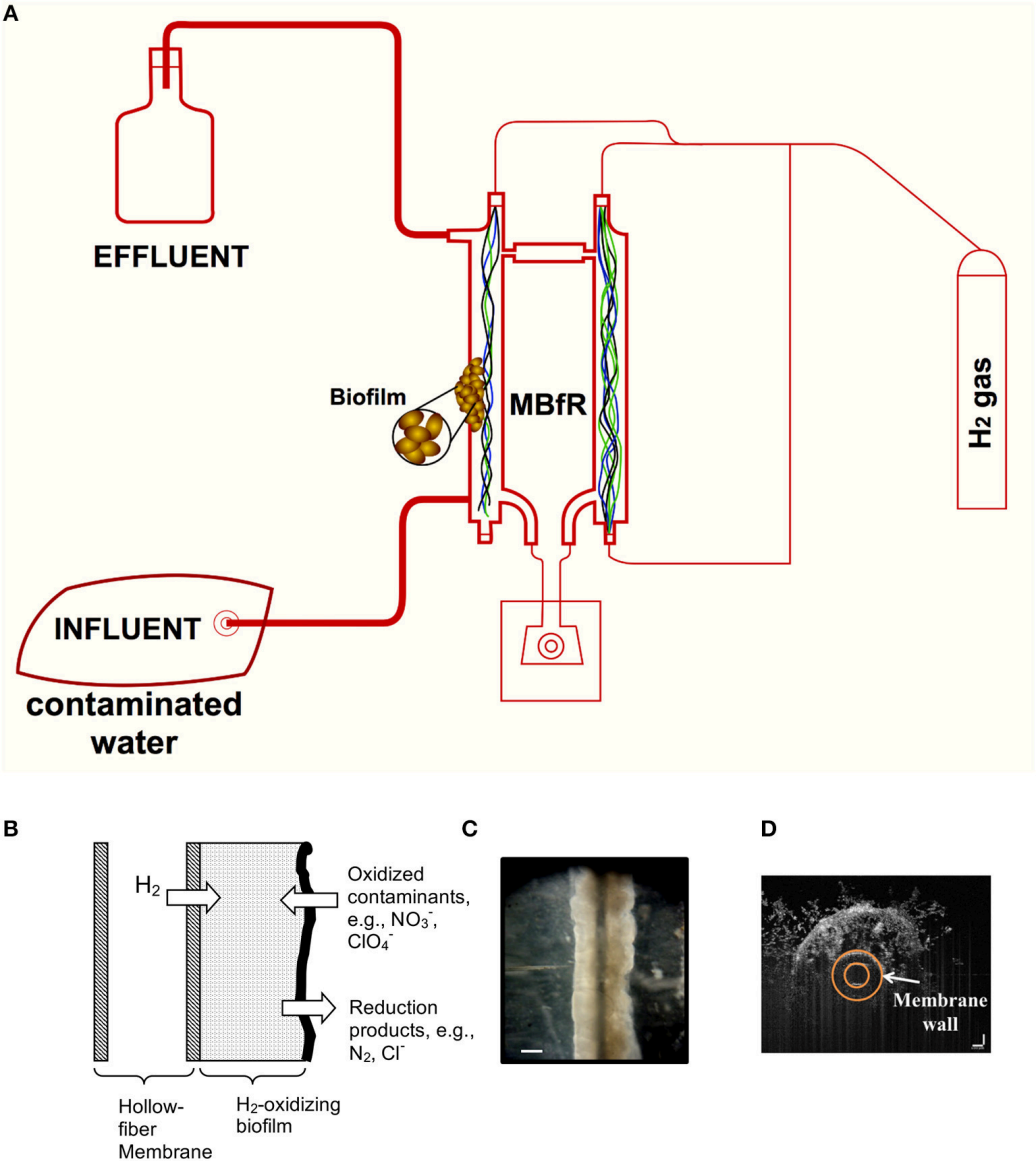
Schematic and images of a bench-scale MBfR system. **(A)** Configuration at the bench-scale, **(B)** biofilm accumulation on the surface of the membranes, **(C)** stereomicrograph of biofilm on 80-μm diameter membrane, and **(D)** optical coherence tomography (OCT) cross-sectional view of biofilm on a 500-μm diameter membrane. Scale bar = 100 μm in **(B,C)**.

A second principle is that the membranes serve as an active substratum for a microbial biofilm community that is composed of microorganisms able to biologically reduce oxyanions in the water (Rittmann, 2018). Figure 1 shows schematically the principles by which the MBfR efficiently and safely delivers H_2_ by diffusion through the membrane wall to a biofilm that accumulates on the membrane's outside surface. The microbial communities in the MBfR biofilms are naturally occurring consortia. Changes in operating conditions (explained later) cause the communities to respond in terms of total accumulation and ecological composition.

### The MBfR Has a Counter-Diffusional Biofilm

**Figure 2:**
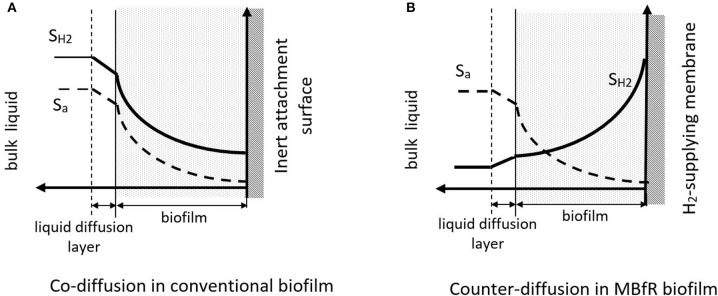
Electron donor and acceptor profiles in **(A)** a conventional, co-diffusional biofilm and **(B)** a counter-diffusional, H_2_-supplying MBfR biofilm. S_H2_ indicates the H_2_ profile, S_a_ indicates the electron acceptor profile. Adapted from Nerenberg (2016).

Biofilms on H_2_-supplying membranes are called counter-diffusional biofilms, because the electron donor and acceptor penetrate the biofilm from opposite sides. This is illustrated in Figure 2. Counter-diffusional biofilms have special characteristics compared to conventional, co-diffusional biofilms, where the donor and acceptor diffuse from the same side of the biofilm. When H_2_ and the oxyanion are supplied from the bulk (co-diffusional biofilm), the most metabolically active region of the biofilm is the exterior, where H_2_ and the electron acceptor substrate are at their highest concentrations. In contrast, in a counter-diffusional biofilm the most active zone may be anywhere within the biofilm, depending on the donor and acceptor concentrations in the biofilm. Counter diffusion of donor and acceptor leads to unique behaviors: development of unique microbial community structures, greater sensitivity to biofilm accumulation, and reduced susceptibility to liquid diffusion layer (LDL) resistance (Nerenberg, 2016). Each is explained below.

#### Development of Unique Microbial Community Structures

When H_2_ is supplied from the base of the biofilm and O_2_ and NO${}_{3}^{-}$ from the bulk, opportunities increase for O_2_-sensitive microbes such as sulfate- and selenate-reducing bacteria, dechlorinating *Dehalococcoides*, acetogens, and methanogens to proliferate near the base of the biofilm. This occurs because the base of the MBfR biofilm has low concentrations of O_2_ and NO${}_{3}^{-}$, but a high concentration of H_2_ (Martin et al., 2015).

#### Greater Sensitivity to Biofilm Accumulation

In all biofilm processes, sufficient biofilm accumulation is needed to obtain high contaminant removal fluxes. In conventional biofilms, fluxes increase with biofilm thickness, but eventually reach a plateau. This occurs because, for a given bulk substrate concentration, the additional active biomass in the outer biofilm is offset by additional inactive biomass in the inner biofilm. Counter-diffusional biofilms behave similarly at low biofilm thicknesses, but excessive thicknesses can lower fluxes. When the biofilm is thick, the greater separation of donor (supplied from the interior) and acceptor (supplied from the bulk liquid) leads to dual limitation where both substrates meet (Nerenberg, 2016). Thus, control of biofilm accumulation is more critical for the MBfR than it is for conventional biofilm processes.

#### Lower Susceptibility to Liquid Diffusion-Layer Resistance

In a conventional biofilm, the liquid diffusion layer (LDL) slows substrate fluxes into the biofilm. As the biofilm thickness and flux increase, the LDL provides mass-transfer resistance that limits further biofilm growth. Higher bulk substrate concentrations are needed to overcome the LDL resistance. In contrast, the LDL in a counter-diffusional biofilm provides a barrier to loss of the internally supplied substrate (e.g., H_2_) to the bulk liquid. Thus, as long as the substrate from the bulk is present at non-rate-limiting concentrations, the LDL will not limit, and it may actually enhance microbial activity (Martin and Nerenberg, 2012).

### The Technology—From Bench to Commercial Scale

**Figure 3:**
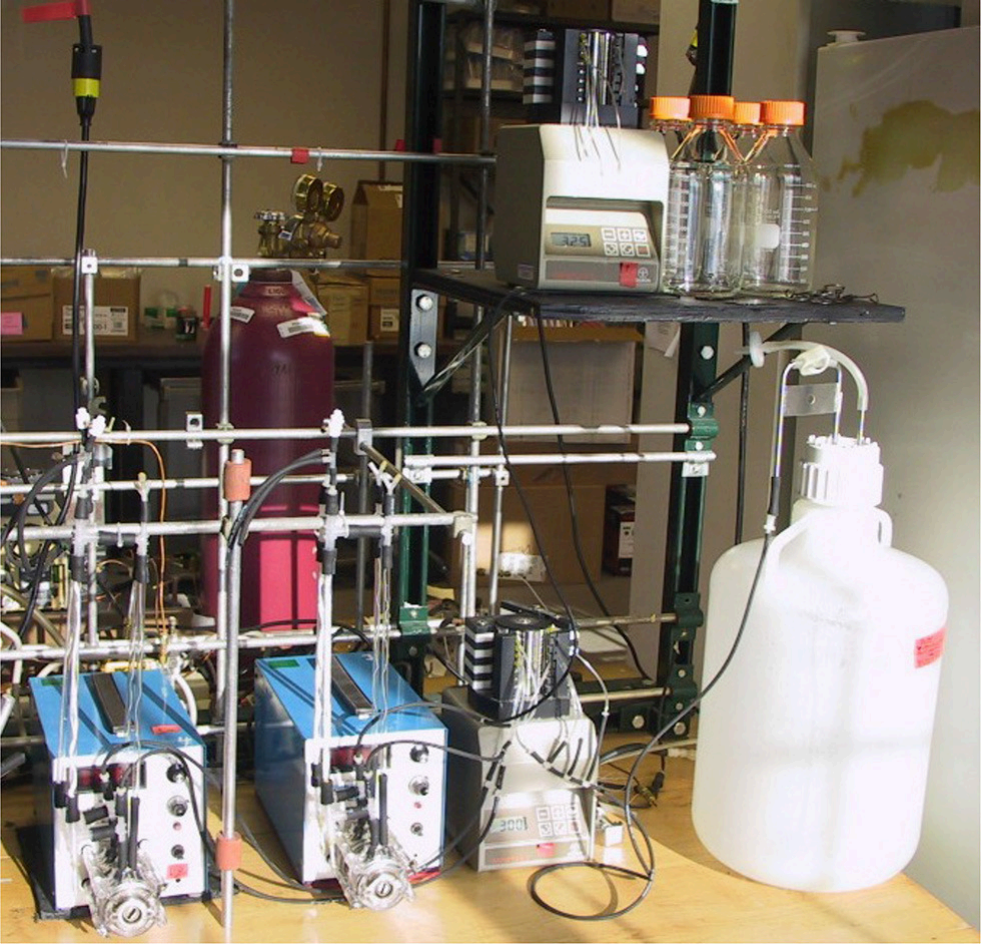
Set of four bench-scale H_2_-MBfRs for nitrate and perchlorate removal. Each reactor consists of a bundle of 32 hollow-fiber membranes in an 8-mm OD glass tube, along with an additional single hollow fiber in a separate tube. Recirculation pumps keep the system well-mixed.

The MBfR technology began in the laboratory, where tests were conducted with a variety of membrane materials, reactor configurations, and water characteristics (Lee and Rittmann, 2002). Most of the MBfR laboratory experiments, as exemplified in Figure 3, were performed with an unstructured, loose bundle of gas-permeable hollow fibers inside an outer tube; the water flow was along the axial length of the fiber bundle. While this configuration has been effective for assessing process feasibility and microbiology, it was not easily scaled up to larger devices required for municipal and industrial applications.

Scale-up and commercial development of the H_2_-based MBfR began in 2005 by Applied Process Technology, Inc., now APTwater, LLC. Numerous pilot and demonstration tests (summarized in Table 1) were conducted to develop methods of fiber handling and to improve biofilm exposure, fluid flow distribution, and biomass management. APTwater had to define large-scale manufacturing techniques and create component supply chains. To reduce market confusion with membrane filtration processes, APTwater adopted the name ARoNite™ for the *autotrophic reduction of nitrate*, and similar ARo names are used for other oxyanions (e.g., ARoPerc for perchlorate reduction).

**Table 1:** Examples of MBfR pilot and demonstration testing.

**Location**	**Contaminant(s)**	**Dates**	**System type**	**Significant outcome**
La Puente, CA	Groundwater ClO${}_{4}^{-}$	~2003	Pilot module	Water Research Foundation (WRF) Report, early system-design information
Modesto, CA	Groundwater NO${}_{3}^{-}$	9/06–6/11	Pilot module	Multiple fiber and module construction improvements, California regulatory approval data collected
Lake Arrowhead, CA	Tertiary effluent NO${}_{3}^{-}$	3/07–11/07	Pilot module	WateReuse Report by Trussell Technologies
San Bernardino, CA	Groundwater NO${}_{3}^{-}$, ClO${}_{4}^{-}$	3/08–1/09	Pilot module	Flow maldistribution limits performance, improvements identified, subsequent project in Rialto authorized with US Dept. of Defense
Glendale, AZ	Groundwater NO${}_{3}^{-}$	4/08–2/09	Pilot module	WRF Report by CH2M-Hill, positive comparison to ion exchange and heterotrophic systems
Rancho Cordova, CA	Groundwater ClO${}_{4}^{-}$	9/08–11/10	Pilot module	Successfully treat 14 ppm to < 4 ppb
Rancho Cordova, CA	Groundwater ClO${}_{4}^{-}$	10/08–11/10	Commercial module	Develop and test larger modules
Ojai, CA	Tertiary effluent NO${}_{3}^{-}$	2/10–12/10	Commercial module	Tested multitude of large modules in one system
Rialto, CA	Groundwater NO${}_{3}^{-}$, ClO${}_{4}^{-}$	5/11–2/12	Commercial module	US Dept. of Defense project with CDM-Smith based on improvements in commercial module
Burbank, CA	Groundwater NO${}_{3}^{-}$, Cr(VI)	6/11–11/12	Commercial module	Tested low ppb Cr(VI) removal
Rancho Cucamonga, CA	Groundwater NO${}_{3}^{-}$	11/11–1/13	Commercial module	1st commercial-scale system, gained regulatory approval for drinking water treatment in California

**Figure 4:**
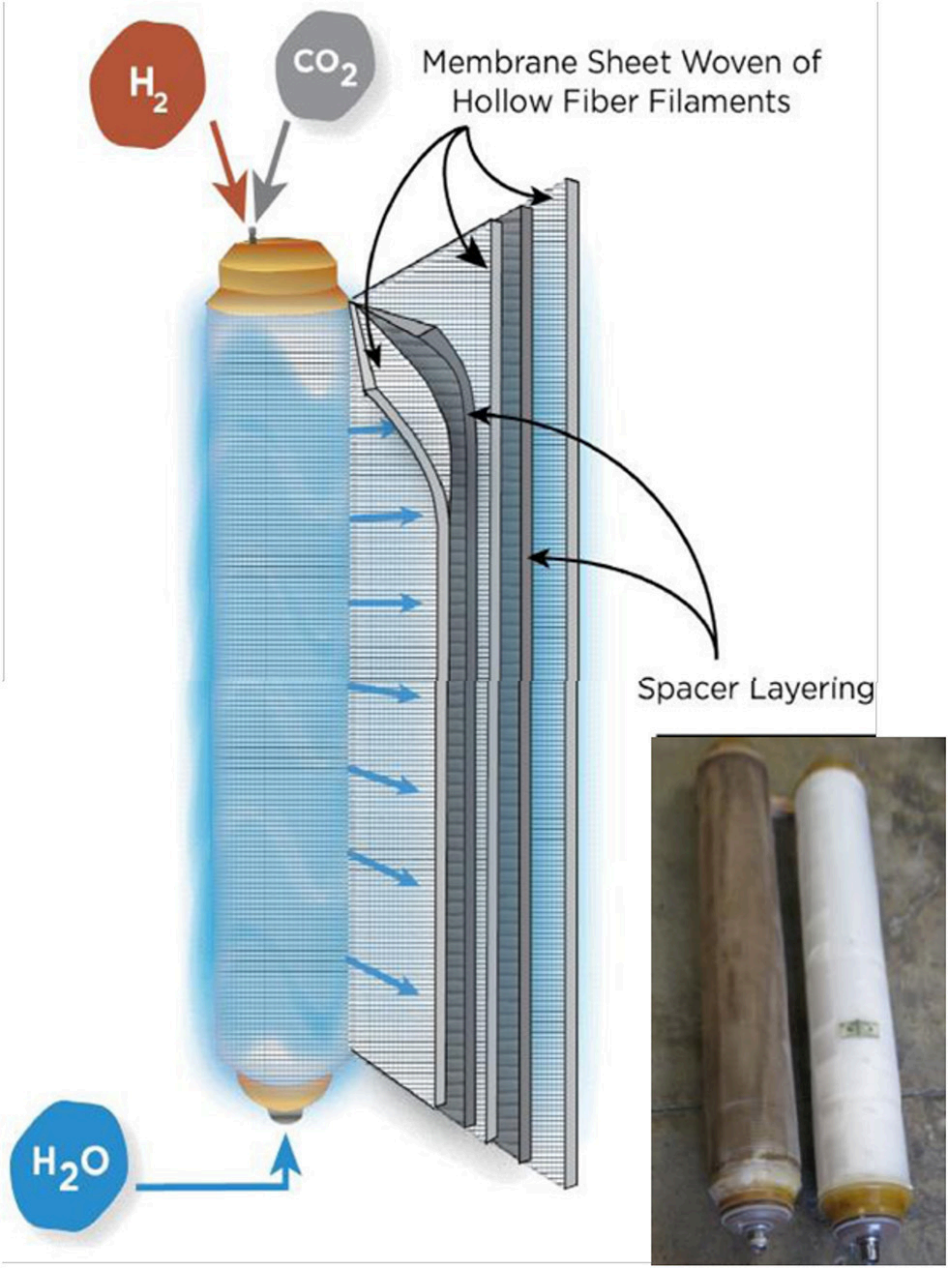
Configuration of Commercial MBfR Module with photo of new and used modules.

Commercial-scale MBfR modules are manufactured by APTwater by weaving gas-permeable hollow fibers into a fabric sheet, several of which are wound onto a perforated core tube along with alternating layers of a spacer mesh which provide fabric sheet separation and water flow distribution. A depiction of the module assembly is shown in Figure 4. Each end of the fabric and spacer assembly is potted in resin and machined to open the lumen ends of each hollow fiber. A cap is placed on each end to provide H_2_ to the lumen of the fibers. Water to be treated is fed into the perforated core tube and travels spirally outward across the surface of the hollow-fiber fabric. Treated water exiting the module can be partially recycled to the module, with the remaining water forwarded downstream.

**Figure 5:**
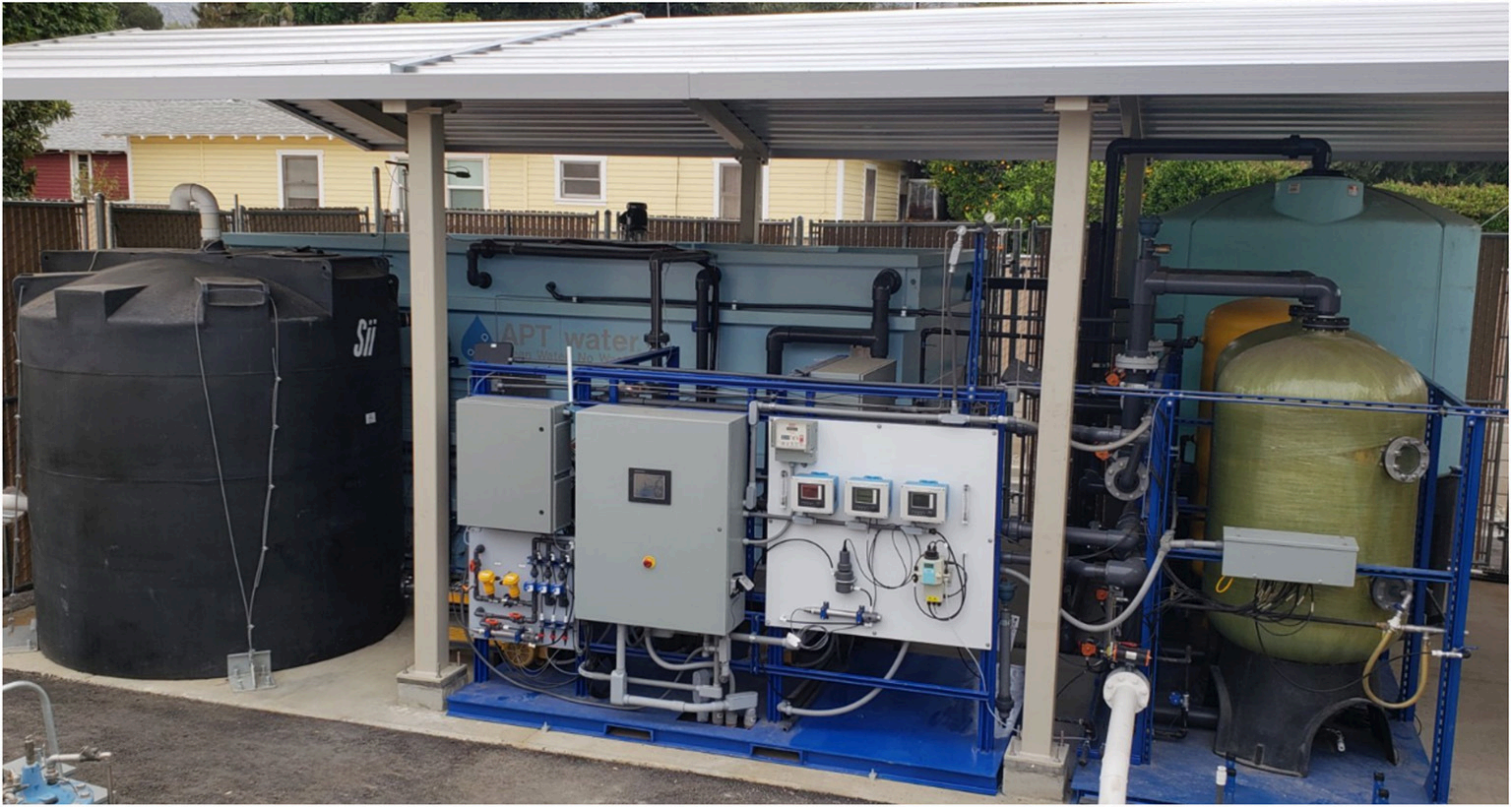
ARoNite™ System in La Crescenta, California.

Approval of the ARoNite™ system by the State of California for municipal water treatment for NO${}_{3}^{-}$ was received in 2013. The first full-scale system was constructed in mid-2018 at a municipal well-site in La Crescenta, California, with startup and commissioning completed in August 2018. After validation testing, it is expected to be in full operation by early 2019. Figure 5 shows a photograph of the latest commercial system.

To compare the MBfR with other existing technologies, a triple-bottom-line analysis was conducted to evaluate life-cycle costs and non-monetary benefits for three nitrate-removal approaches in the context of a 10 million gallons/day groundwater treatment facility for the City of Glendale, Arizona (Meyer et al., 2010). The three approaches were: (1) MBfR + Ozone + Biologically Active Carbon Filtration, (2) Static Upflow Bed with Plastic Media + Ozone + Biologically Active Carbon Filtration (a conventional biological nitrate removal approach), and (3) Fixed Bed Ion Exchange (a conventional physicochemical approach). Compared to the second approach, the first approach provided more environmental benefits due to better water utilization, more drinking water quality benefits due to better contaminant removal, and more residuals benefits due to less production of solids/biomass. However, the second approach represented a slightly lower total cost and offered more societal benefits due to less maintenance complexity. The third approach had the highest score for permitting, since it has already been widely applied, but the poorest scores for societal and environmental impacts due to the production of large quantity of residues. The cost of the third approach was between the first two approaches.

## Current challenges of the technology

### Biofilm Management

A biofilm's capacity to reduce oxyanions depends on the biofilm accumulation and activity. Accumulation is the total mass of biofilm per unit surface area, and it is the product of the biofilm's thickness and biomass density. As discussed previously, a biofilm that is too thin has inadequate biomass to catalyze the reaction, but a biofilm that is too thick increases the mass transfer resistance into the biofilm. Biofilm accumulation can be managed in part via a loop that recirculates part of the treated water back into the MBfR, because the recirculation rate affects biofilm detachment through shear stress and turbulence (Rittman, 1982; Cunningham et al., 1991). Biofilm accumulation also is managed by periodic (e.g., daily) high-shear events, in which excess biomass is detached (usually by a gas sparge to create a two-phase gas-water high velocity stream) and then removed from the reactor (Evans et al., 2013).

Biofilm activity is affected by the rate of detachment. Minimal detachment allows non-active biomass to accumulate in the biofilm, and this slows the biofilm's metabolic activity. Regular detachment keeps the biofilm more metabolically active.

Biofilm activity is also affected by the pH, which usually increases in the MBfR used for oxyanion reduction due to proton consumption. Table 2 summarizes the biological reactions for H_2_ and key oxyanions. The molar proton consumption per mole of oxyanion varies from 0.11 to 2, depending on the oxyanion. Too much pH increase not only can inhibit the microorganisms' activity, but also can lead to precipitation of minerals, such as calcium carbonate, calcium hydrogen phosphate, calcium dihydrogen phosphate, hydroxyapatite, and β-tricalcium phosphate (Lee and Rittmann, 2003). The minerals in the biofilm increases mass transfer resistance, which makes the biofilm less active. The pH increase and precipitation can be quantified by a model developed based on mass and charge balance (Tang et al., 2011). The pH can be controlled by two methods. The first method is adding acid (e.g., HCl) into the influent at a concentration that balances the proton consumption, and the second method is sparging carbon dioxide into the reactor at a set point using a pH-control loop (Evans et al., 2013). The model in Tang et al. (2011) predicts the amount of acid needed in the first method and the pH set point in the second method. The second method is implemented at the commercial scale using pre-mixed gas containing CO_2_.

**Table 2:** Biological reactions involving H_2_ oxidation and oxyanion reduction.

**Reaction**	**References**
NO${}_{3}^{-}$+ 3.0H_2_ + 0.23CO_2_ + H^+^ = 0.48N_2_ + 0.046C_5_H_7_O_2_N + 3.4H_2_O	Lai et al., 2014
NO${}_{2}^{-}$+ 1.8H_2_ + 0.12CO_2_ + H^+^ = 0.49N_2_ + 0.024C_5_H_7_O_2_N + 2.2H_2_O	Tang et al., 2011
ClO${}_{4}^{-}$+ 5.5H_2_ + 0.53CO_2_ + 0.11NO${}_{3}^{-}$+ 0.11H^+^ = Cl^−^+ 0.11C_5_H_7_O_2_N + 5.2H_2_O	Zhao et al., 2011
SeO${}_{4}^{2-}$+ 3.4H_2_ + 0.13CO_2_ + 0.027NO${}_{3}^{-}$+ 2.0H^+^ = Se +0.027C_5_H_7_O_2_N + 4.3H_2_O	Lai et al., 2014
SO${}_{4}^{2-}$+ 4.2H_2_ + 0.075CO_2_ + 0.015NO${}_{3}^{-}$+ 1.5H^+^ = 0.5H_2_S + 0.5 HS^−^+ 0.015C_5_H_7_O_2_N + 4.2 H_2_O	Rittmann and McCarty, 2001

### Gas Back-Diffusion in Hollow-Fiber Membranes

When H_2_ gas is supplied via hollow-fiber membranes, other dissolved gases in the bulk liquid or gases formed within the biofilm can diffuse into the membrane, diluting the H_2_ gas. When operating an MBfR with membranes sealed on one end, these gases concentrate at the distal end of the membrane, possibly decreasing their effectiveness for H_2_ transfer and leading to thinner biofilms (Ahmed et al., 2004; Perez-Calleja et al., 2017).

Operating with open-ended membranes eliminates this problem, but leads to loss of H_2_, which wastes H_2_ and also creates a potential combustion hazard. A better solution is to periodically vent the membrane. By opening the sealed end for a few seconds every few minutes, the H_2_ level can be re-established within the membrane with minimal waste (Perez-Calleja et al., 2017).

### Biofilm Microbial Ecology

**Figure 6:**
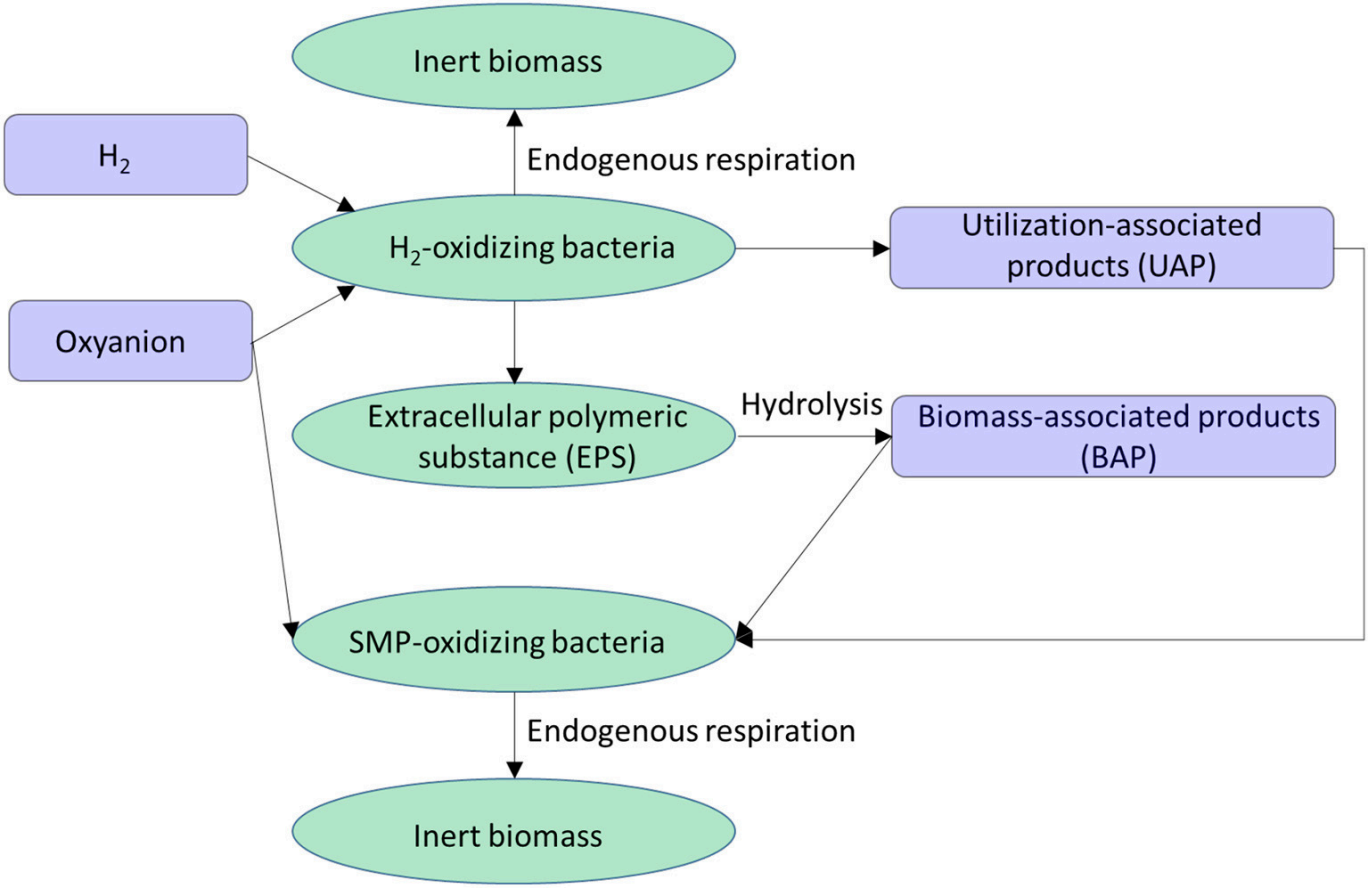
Interaction of the four biomass species (ellipses) via four dissolved chemical species (rectangles) in a H_2_-based biofilm.

A biofilm in the H_2_-based MBfR used for removing an oxyanion usually consists of four biomass types: (1) H_2_-oxidizing bacteria, (2) SMP (soluble microbial products)-oxidizing bacteria, (3) extracellular polymeric substances (EPS), and (4) inert biomass. As illustrated in Figure 6, the four biomass types interact with each other through four dissolved chemical species: H_2_, the oxyanion, utilization-associated products (UAP), and biomass-associated products (BAP) (Laspidou and Rittmann, 2004; Tang et al., 2012a; Liu et al., 2016). In brief, the H_2_-oxidizing bacteria use H_2_ to reduce the oxyanion to a harmless or immobilized form, and they produce EPS and UAP as byproducts. The EPS help the microorganisms to stick together and to the membrane surface area. EPS accumulation is balanced by its hydrolysis to BAP. The UAP and BAP together are called SMP, which stimulate the growth of the SMP-oxidizing bacteria that also reduce the oxyanion. Both active biomass species can be converted to inert biomass through endogenous respiration.

The co-existence of several oxyanions is common and adds more interactions among the biomass species and the dissolved chemical species. The added interactions depend on the co-existing oxyanions. When the oxyanions (e.g., nitrate and sulfate) are reduced by different bacteria (Moura et al., 1997), the interactions can be described by including one more kind of H_2_-oxidizing bacteria that use H_2_ in parallel (e.g., H_2_-oxidizing denitrifying bacteria and H_2_-oxidizing sulfate-reducing bacteria) (Tang et al., 2013). The two kinds H_2_-oxidizing bacteria compete for H_2_ and space in the biofilm. Previous experimental and modeling studies have shown that often a subordinate electron acceptor (e.g., sulfate) is not reduced unless the more dominant electron acceptor (e.g., NO${}_{3}^{-}$) surface loading (defined below) is low (Tang et al., 2013).

The co-existence of nitrate and perchlorate represents a more complicated interactive scenario, since most H_2_-oxidizing perchlorate-reducing bacteria also can use nitrate as an electron acceptor (Kengen et al., 1999; Giblin and Frankenberger, 2001; Chaudhuri et al., 2002; Okeke et al., 2002); this leads to competitive inhibition between nitrate and perchlorate on the one hand, but promotion of perchlorate reduction by nitrate on the other hand (Tang et al., 2012a). The interactions are quantified in some case studies in the next section.

To close this section, Table 3 identifies needs for moving the MBfR technology forward for the range of applications in water-quality engineering.

**Table 3:** Areas of opportunity for improving the MBfR technology.

**Criteria**	**Action to take**	**References**
Biofilm management	Several modeling studies and experimental projects constitute the benchmark of the technology. For the larger scale application, it is necessary to keep focusing on several aspects such as microbial ecology, biofilm detachment, gradients within the biofilm and others	Martin and Nerenberg, 2012
Quantify environmental footprint	Perform holistic sustainability analysis of the technology. An example could be a Life Cycle Analysis	Meyer et al., 2010 Evans et al., 2013
Thorough economic evaluation	It has been reported previously for some of the pilot studies. But, a more comprehensive assessment is indeed needed to even report on the social impacts of the technology	Meyer et al., 2010 Evans et al., 2013
Combination with other technologies	This area can be explored for the treatment of complex case studies such as those with several contaminants	Nerenberg, 2016
Resource recovery of valuable compounds	Palladium and elemental selenium has been precipitated and accumulated in the biofilm as results of oxyanion reductions. However, further research on the direction of resource recovery is necessary	Zhou et al., 2016 Ontiveros-Valencia et al., 2016

## Performance Case Studies

The MBfR has been tested for the treatment of many oxyanions: e.g., nitrate (Lee and Rittmann, 2002), perchlorate (Nerenberg et al., 2002), chromate (Chung et al., 2006a; Lai et al., 2016; Long et al., 2017), selenate (Chung et al., 2006b; Van Ginkel et al., 2011b; Ontiveros-Valencia et al., 2016), hexavalent uranium (Zhou et al., 2014; Ontiveros-Valencia et al., 2017), and chlorinated solvents (Chung et al., 2007a; Chung and Rittmann, 2008; Ziv-El et al., 2012; Long et al., 2018). The MBfR is versatile and can be adjusted for the treatment of a single oxyanion or a mixture of them. It also is able to treat high concentrations of water contaminants (Ontiveros-Valencia et al., 2013; Zhou et al., 2014), even in brines from ion exchange columns (Chung et al., 2007b; Van Ginkel et al., 2008).

This section first lays out the criteria needed to define MBfR performance for reducing oxyanions. It then provides a series of case studies that demonstrate that the MBfR can reduce one or several oxyanions and that show how to use the performance criteria to achieve treatment goals.

### Performance Criteria

The performance of an MBfR can be established using four criteria: effluent concentration, percentage removal, surface loading, and removal flux. Each is defined here and used to characterize a set of case studies that follow. An oxyanion's effluent concentration (denoted *C*_eff_) determines whether or not the treatment goal has been attained. *C*_eff_ has concentration units, or M/L^3^. *C*_eff_ can be compared to an MCL or discharge standard. The percentage removal (%Rem) compares *C*_eff_ to the influent concentration, *C*_inf_:

(9) $$\%REM=\;100\%\;\frac{C_{\inf}-\;C_{eff}}{C_{\inf}}$$

The surface loading (*SL*) is

(10) $$\%REM=\;100\%\;\frac{C_{\inf}-\;C_{eff}}{C_{\inf}}$$

in which *SL* has units of M/L^2^-T, *Q* is the influent flow rate (L^3^/T), and *A* is the biofilm surface area (L^2^). The surface loading is used to define the size of the MBfR in terms of its biofilm surface area, or *A* = *QC*_inf_ /SL. As MBfR modules have a set area, computing *A* from the *SL* makes it possible to determine the number of modules, which leads to estimate of the capital costs and areal footprint. Finally, the removal flux (J, also in units of M/L^2^-T) is

(11) $$J=\;\frac{Q\;(C_{\inf}-\;C_{eff})}{A}=SL\;(\frac{\%REM}{100\%})$$

The removal flux is the design parameter for achieving the desired *C*_eff_ for a given oxyanion and for mediating competition among different electron acceptors (Ziv-El and Rittmann, 2009; Zhao et al., 2013a,b; Ontiveros-Valencia et al., 2014a). Ideally, a study provides all of the performance criteria, and this makes it possible to assess mechanisms, regulatory compliance, and economics.

### Nitrate Alone—Groundwater and Wastewater Secondary Effluent

The performance of the H_2_-based MBfR for denitrification has been summarized previously by Di Capua et al. (2015) and Karanasios et al. (2010). It is common to report MBfR denitrification performance in terms of removal fluxes. However, it is important to note that removal fluxes can vary significantly for the same reactor, depending on the H_2_-supply pressure, the type of membrane, whether the membranes are operated as open ended or closed ended, the bulk nitrate concentration, the biofilm thickness, the type of membrane material, the mixing regime, and the water temperature.

Reported denitrification fluxes ranged from <0.3 g N m^−2^ d^−1^ to over 14 g N m^−2^ d^−1^, but were commonly between 0.5 and 2 g N m^−2^ d^−1^. Higher fluxes tended to occur with high influent loadings, higher H_2_ supply pressures, and batch operation, which allows higher bulk nitrate concentrations for much of the treatment cycle. Percent removals from 90 to 99 percent are common. In practice, the nitrate flux and loading are usually controlled by the effluent concentration of nitrite (a nitrate-reduction daughter product), rather than the nitrate concentration, since the maximum contaminant level (MCL) set by USEPA is 10 times lower for nitrite (1 mg N/L) than for nitrate (10 mg N/L) (USEPA, 2012). For example, in a pilot-scale study conducted by Tang et al. (2010), the effluent nitrite concentration reached the MCL of 1 mg N/L at a nitrate surface loading rate of 6 g N/m^−2^ d^−1^, when the effluent nitrate concentration was below its MCL of 10 mg N/L.

### Nitrate and Perchlorate in Groundwater

The simultaneous removal of nitrate and perchlorate was studied experimentally and via modeling (Zhao et al., 2011; Tang et al., 2012a,b). Nitrate affects perchlorate removal in two opposing ways. On the one hand, nitrate promotes perchlorate removal by stimulating the perchlorate-reducing bacteria, since most perchlorate-reducing bacteria can use nitrate as the primary electron acceptor. On the other hand, nitrate can inhibit perchlorate by competing for common resources, such as H_2_ and space in the biofilm. A multispecies biofilm model based on these mechanisms was developed in Tang et al. (2012a,b) and validated by experimental results in Zhao et al. (2011). The model predicted that nitrate promotes perchlorate reduction when the nitrate loading is <0.1 g N/m^2^-day, because the promotion mechanism dominates; however, it inhibits perchlorate reduction when the nitrate loading is higher than 0.6 g N/m^2^-day, because the inhibition mechanism dominates. Nitrate's effect on perchlorate reduction is negligible when the nitrate loading rate is between 0.1 and 0.6 g N/m^2^-day. Perchlorate can be removed to a few ppb level when the nitrate loading is below 0.6 g N/m^2^-day and the perchlorate loading is below 0.01 g ClO${}_{4}^{-}$/m^2^-day (Tang et al., 2012b). In practice, the influent nitrate concentration usually is a few orders of magnitude higher than the influent perchlorate concentration (Kimbrough and Parekh, 2007). In this situation, a two-stage MBfR can be used (Zhao et al., 2013b; Ontiveros-Valencia et al., 2014b). The first stage can remove nitrate at a very high loading rate up to 6 g N/m^2^-day (Tang et al., 2010), and the second stage can remove both nitrate and perchlorate simultaneously at a nitrate loading rate lower than 0.6 g N/m^2^-day.

### Nitrate, Sulfate, and Selenate in Flue-Gas-Desulfurization or Mining Wastewater

Flue-gas desulfurization (FGD) and coal mining are two of the major sources of selenium in the environment. FGD is used to remove sulfur dioxide from exhaust gases of power plants, and FGD wastewaters have high concentrations of total dissolved solids (TDS), nitrate, nitrite, sulfate, and selenate. Despite the challenging conditions that FGD brines present, they have been successfully treated in the MBfR (Van Ginkel et al., 2010, 2011a,b). In those studies, high TDS concentrations (e.g., 15–33 g/L) did not interfere with the reduction of nitrate and selenate by the biofilm microbial community. Furthermore, sulfate reduction did not occur in the above-mentioned studies. Microbial competition for electron donor (H_2_ gas) and the high TDS levels seemed to be related with the lack of sulfate reduction when denitrification and selenate reduction were the dominant and subordinate microbial processes, respectively (Van Ginkel et al., 2010, 2011a,b). Nitrate at 500 mg N/L and selenate at 36 mg/L were reduced up to 98 and 99%, respectively, in synthetic FGD brines. Experimentation with real FGD brines (nitrate/nitrite concentration of 56 mg N/L and 10 mg/L of selenate) also achieved the same reduction rates observed with synthetic FGD. Thus, the MBfR achieved a total-Se concentration in the ppb range (Van Ginkel et al., 2011b). It appears that the co-reductions of nitrate/nitrite and selenate increased biofilm accumulation and improved the removal of both oxyanions.

Because denitrification and selenate reduction can raise the pH, it was necessary to control the pH to avoid precipitation of calcium and magnesium ions from the brines. A pH of 6.8 in the bulk liquid was sufficient to avoid an excessively high pH inside of the biofilm, and, consequently, the reduction rates for nitrate and selenate were not affected (Van Ginkel et al., 2011a).

A synthetic mining wastewater, contained up to 45 mg N/L nitrate and selenate of 0.6 mg/L, was treated in a two-stage MBfR system. The first stage, called lead MBfR, reduced the concentration of the dominant oxyanion (Ontiveros-Valencia et al., 2014a), nitrate in this case. The second stage, called the lag MBfR, finished the treatment by taking the concentration of the sub-ordinate oxyanion, selenate for this study, to a low concentration. The lead MBfR was responsible of average nitrate reduction rate of 91–94%, and the lag MBfR achieved selenate reduction rates up to 87% (Mehta, 2016). Depending on the flow rate at which the lead and lag MBfRs were operated, the range of selenate surface loadings was 0.004–0.04 g Se/m^2^ day, while the nitrate loading range was 0.1–1 g N m^−2^ d^−1^.

Not only is the MBfR capable of removing selenate from FGD brines and mining wastewater, but it also produces and retains elemental selenium (Se^0^) in the biofilm matrix. Transmission electron microscopy coupled with energy dispersive x-ray spectroscopy (TEM-EDX) analysis of the solids from the MBfR biofilm documented the presence of elemental selenium (Ontiveros-Valencia et al., 2016). Thus, the MBfR holds promise to recover a valuable resource.

### Metal Oxyanions: Chromate as an Example

Heavy metal contamination of the environment is a widely recognized concern. Oxidized metals in the form of oxide anions are very soluble, which leads to contaminant transport after discharge into aqueous streams and threatens ecological function and human health. For instance, hexavalent chromium [Cr(VI)], present as soluble chromate ion (CrO${}_{4}^{2-}$), causes irritation of the skin, eyes, and mucous membranes (Leonard and Lauwerys, 1980).

Some of these toxic metal oxyanions can be reductively transformed by microorganisms into less toxic and/or insoluble forms. In the example of chromium, bacteria utilizing H_2_ as the electron donor, reduce the hexavalent chromate anion to the trivalent chromite (Cr^3+^) cation:

(12) $${\text{CrO}}_{4}^{2-}+1.5{\text{H}}_{2}+5{\text{H}}^{+}\to {\text{Cr}}^{3+}+4{\text{H}}_{2}\text{O}$$

In neutral or basic conditions (pH > 6), Cr^3+^ spontaneously precipitates with OH^−^:

(13) $$\text{C}r^{3+}+3{\text{OH}}^{-}⇌\text{Cr}{\left(\text{OH}\right)}_{3\left(s\right)}$$

The Cr(OH)_3_ precipitate tends to associate with bacterial cell surfaces and extracellular polymeric substances (EPS), particularly in biofilm matrices, and it is consequently separated and immobilized from water.

A number of MBfR studies reported successful bio-reduction of Cr(VI) as the sole electron acceptor(Long et al., 2017; Zhong et al., 2017) or along with primary electron acceptors including nitrate, sulfate, and/or selenate (Chung et al., 2006c, 2007c; Lv et al., 2017). The Cr-only tests support that the biofilms were able to grow by respiring Cr(VI), and Cr(VI) reduction was not only a detoxification response against oxidative stress (Sedláček and Kučera, 2010). The multi-acceptor tests reveal that other electron acceptors affected Cr(VI)-reducing capacity in distinct ways. On one hand, substantial nitrate and/or sulfate reduction caused considerable retardation of Cr(VI) removal (Lv et al., 2017). For example, after 10 mg N/L of nitrate was added, the Cr(VI)-reducing flux significantly dropped from 0.12 to 0.08 g Cr(VI)/ m^2^ day, or from 86 to 57% removal of the initial 1 mg Cr(VI)/L (Chung et al., 2006a).

## Conclusion

Bioreduction is an effective means to detoxify waters contaminated with oxyanions. The key to bioreduction is delivering the electron-donor substrate. H_2_ gas is a highly advantageous donor, because it is non-toxic, utilized for bioreductions of the wide range of oxyanions, relatively inexpensive, and not easily over-dosed. The challenge for using H_2_ as the donor is that it has low water solubility and is combustible when mixed with air. The challenges are overcome with the MBfR, in which H_2_ gas is delivered directly to a biofilm that accumulates on the outside of bubbleless gas-transfer membranes. This creates a counter-current biofilm, in which H_2_ diffuses through the biofilm from the membrane substratum, while the oxyanion electron acceptors diffuse through the biofilm from the bulk-liquid side of the biofilm. H_2_ delivery is on-demand, based on the supply rate of electron acceptors, and can be nearly 100% efficient.

The MBfR, a commercially available technology, has well-documented ability to biologically reduce a wide range of oxyanions to harmless products: e.g., nitrate, nitrite, perchlorate, selenate, chromate, and numerous others. In some cases, the reduced products are solids with economic value, such as elemental selenium.

Being a biofilm process, the main design criterion is the surface loading of the oxyanion. The H_2_-delivery capacity, which must be matched to the oxyanion-reduction rate, is readily controlled by the H_2_ pressure to the membrane lumen. Managing the accumulation and activity of the biofilm is important for process success, and means to do so include the water flow velocity past the membrane, periodic high-shear events, and pH control.

## Author Contributions

All authors listed have made a substantial, direct and intellectual contribution to the work, and approved it for publication.

### Conflict of Interest Statement

DF is an employee of APTwater, which is commercializing the MBfR. The remaining authors declare that the research was conducted in the absence of any commercial or financial relationships that could be construed as a potential conflict of interest.
